# Humans, Other Animals and ‘One Health’ in the Early Twenty-First Century

**DOI:** 10.1007/978-3-319-64337-3_6

**Published:** 2017-12-31

**Authors:** Angela Cassidy

**Affiliations:** Department of Politics, University of Exeter Department of Politics, Exeter, UK

## Abstract

This chapter explores the history of recent movements for One Health, which argue that because many of today’s pressing health problems lie at the interface of human, animal and environmental health, they can only be managed effectively by breaking down traditional disciplinary silos. It explores how Schwabe’s work influenced, and was reconfigured by, this movement, and locates its early development in several different research and policy networks, which produced not one but several different forms of One Health. The chapter also examines how human–animal health relationships have inspired and shaped One Health, and how they are represented—in sometimes contradictory ways—in the texts and images produced by One Health researchers and advocates. It argues that in foregrounding the roles of animals as transmitters of diseases to humans, and as experimental models of human disease, One Health rebrands existing longstanding research agendas that are more concerned with the health of humans than that of animals.

Angela Cassidy

In 2002 the US-based Association for Veterinary Epidemiology and Preventive Medicine (AVEPM) co-organized a symposium in St. Louis, Missouri, to honour the lifetime achievements of Calvin W. Schwabe, who had served as professor of epidemiology in both the medical and veterinary schools of the University of California, Davis until his retirement in 1991.^1^ At the symposium, Schwabe gave a keynote address summarizing his ideas about how veterinary medicine should relate to other disciplines—particularly human medicine—and the wider world. Arguing against the compartmentalization of medicine by species, into human and veterinary strands, he used the opportunity to restate his longstanding arguments for a philosophy of One Medicine ( OM), which sees veterinary medicine as a close collaborative partner with human medicine, working together towards the broader endeavours of healthcare and furthering knowledge in the life sciences.^2^ He claimed that this positioning reflected and recognized the contributions that veterinary medicine made to a range of other fields, including comparative zoology, parasitology, epidemiology, human public health, agriculture and conservation.^3^ Schwabe’s philosophy of OM was inspired by and expressed in his own career trajectory, which cut across many of these disciplines, as exemplified by his work on the tapeworm , described in Chapter 10.1007/978-3-319-64337-3_5. He had first written about bringing ‘Veterinary Medicine and Human Health’ together during the early 1960s, while working at the (AUB) University of Beirut and consulting on parasitology for the World Health Organization (WHO). However,﻿ he elaborated his ideas and first described them using the term OM during the 1980s, following his return to the USA.^4^

The other contributors to the 2002 symposium, who included some of Schwabe’s many students and collaborators (now senior academics and policy-makers in their own right), discussed the relevance of OM to their own work in areas such as disease surveillance, veterinary public health and epidemiology.^5^ The event was particularly timely because Schwabe was becoming a figurehead for a wide network of scientists and health professionals working across human, animal and environmental health. While many of these individuals had been grappling with scientific and policy problems lying at the intersection of these fields for some time, Schwabe’s ideas helped them articulate why it was necessary to think in this integrative way. His ideas about OM also provided a key foundation for the later emergence of One Health ( OH), a broader reconfiguration of research, policy and clinical practice across human and animal health, which also brought in environmental concerns.

The term OH first appeared in 2003, when it was adopted by several groups working across human and animal health, and subsequently by policy-makers, clinicians and researchers.^6^ Its initial impetus came from renewed fears about the emergence and spread of zoonotic diseases passing between humans and other animals.^7^ In November 2002, as the veterinary epidemiologists celebrated Schwabe’s career, a previously unidentified coronavirus that originated in an as yet unidentified animal was causing a global outbreak of severe acute respiratory syndrome (SARS), which took six months to contain and killed more than 700 people. This was the first of a series of crises and near-crises related to zoonoses , including the emergence of new strains of highly pathogenic avian influenza (HPAI ) in the mid-2000s. Such events refocused scientific and policy attention on the transmission of infectious diseases from animal to human populations.^8^ They also brought wider recognition of the problems posed by the traditional separation of human and animal health in science, policy and the professions, particularly when (as in the case of HPAI) they created ‘ silos’ that limited the ability to share knowledge or coordinate policy across international health organizations.^9^ This situation resulted in calls for more effective and integrated working across these domains.

Since 2010, OH ideas have spread across the world, with research and advocacy groups forming in, for example, South Korea, Japan, Sweden and Australia. Postgraduate courses in OH have been launched in several countries, while in the USA, universities are experimenting with joint teaching across medical and veterinary schools. Alongside this institutionalization, the scientific literature has matured, with journal citation rates shooting up, two textbooks being published in 2014^10^ and new journals being launched in 2011 and 2015.^11^ Within this literature, Schwabe is widely referenced as a key source of its ideas, a visionary who coined the term OM,^12^ and whose career established him as the ‘father of veterinary epidemiology’.^13^

This chapter turns the spotlight on the recent history of OH as a self-conscious movement in the twenty-first century, analysing its emergence and the roles that Calvin Schwabe played in it. While it is a more human-centred chapter than the others in this volume, it follows them in demonstrating the zoological foundations of medicine by examining the human–animal health relations that underpin the OH movement. The first half identifies Schwabe as a key source of ideas and inspiration, as well as the latest in a series of historical progenitors for OH today. It documents how the idea of OH was developed by different academics, clinicians and policy-makers working in specific institutional contexts to produce not one but many OHs, which awarded different roles to animals, offered different portrayals of their health relationships with humans, presented multiple interpretations of the term ‘zoonosis’ and held different visions of how disciplinary relationships needed to change in order to achieve the desired integration of human and animal health. 14 It argues that, like the earlier intersections of human and animal health and medicine explored in the other chapters, OH is a response mounted by specific researchers (and policymakers) to problems manifesting at particular times and in particular places. In contrast to advocates’ claims, it is not a self-evidently beneficial phenomenon, nor the result of inevitable progress, but a contingent and context-bound activity that is actively and continually created through persuasive rhetoric and alliance-building.^15^

The second half of the chapter focuses on the animal subjects of OH. It asks what sorts of animal feature in the images and scientific literatures associated with OH, and in what types of roles and relationships with humans. Where in the world do they come from? How are they perceived in relation to each other and to humans, and what can this tell us about the relative prioritization of human and animal health within the OH agenda?^16^ As in earlier chapters, this analysis involves the scrutiny of traces left by animals on the medical historical record.^17^ The photographs, infographics and logos used by advocates as they make their case for OH offer a particularly distinctive type of animal trace that—like other cultural material, such as films, photographs, artistic portrayals, fictions, illustrations, advertising and even clipart—provide a rich source of information about the roles that animals play in medicine and society.^18^ Such sources have been used previously to investigate human–animal relationships, and to illuminate how human recipients of care are represented and understood, particularly in health and international development contexts.^19^ While the symbolic representations of animals that they contain constitute less direct animal traces than those in the scientific and other sources analysed elsewhere in this volume, they are traces nonetheless that are left on and remade by the human imagination. They might differ wildly from animals themselves, up to and including completely imaginary animals, but it is unlikely that humans could create these images without encountering animals in the first place. Therefore their analysis can offer meaningful insights into human– animal relationships. In the case of OH, they are all the more important because they are created and used with the intention of shaping how humans interact with animals in the future.

Drawing on these analyses of animal images, and the imagery used in campaigns for public health, global health and international development, the second half of the chapter identifies the contradictions inherent in the OH portrayal of animals, the implications for the trajectories of OH research and practice, and for the health of the humans and animals on which they are projected to impact. Distinguishing between the portrayal of ‘animals’ as a generic category and as specific living beings, it will argue that animals feature in OH primarily in roles that either threaten or promise to advance human health, such as transmitters of infectious disease, sources of nutrition and companionship, and experimental models for the advancement of medical science and technology. In contrast to the case studies presented in some of the earlier chapters, which similarly examined the intertwining of human and animal health, knowledge about and concern for animals in their own right does not appear to be a major topic of interest in OH. Despite the stated aim—to bring human and animal health closer together—this substantive focus on ultimately advancing human health may create a paradoxical situation where OH advocacy ends up reinforcing the very anthropocentrism that it seeks to change.

## One Health or Many?

The One Health concept is a worldwide strategy for expanding interdisciplinary collaborations and communications in all aspects of health care for humans, animals and the environment.^20^

**Fig. 6.1:**
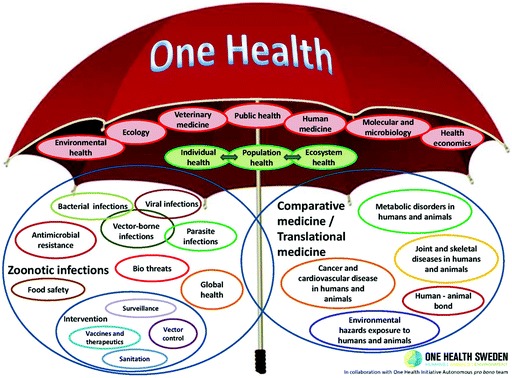
The One Health umbrella. *Source* OH Sweden, 2014

This definition of OH was developed by the OH Initiative, a US-based advocacy group: an unfunded group of public health physicians and veterinarians in favour of human–animal health collaboration.^21^ While useful for advocates, this definition has not gone uncontested, and the broad and flexible nature of the OH concept has been widely debated. While some believe that this breadth risks losing all meaning and has been detrimental to implementing ideas in practice,^22^ others have argued that OH acts as a usefully flexible ‘boundary object’ or ‘ umbrella’ under which a range of topics, disciplines and forms of collaboration can shelter, facilitating interdisciplinary cooperation up to and including the social sciences and humanities.^23^ The metaphor of OH as an umbrella (originally formulated by policy analyst Aline Leboeuf) has proved to be popular among OH advocates. Fig. 6.1 shows a graphic created by One Health Sweden, depicting fields dealing with zoonosis sheltering on one side, and those involved in clinical research and practice on the other.^24^ While this metaphor evidently helps OH advocates to articulate both the breadth and the limits of their endeavour, it abstracts the idea away from the personal, practical and institutional contexts where it originated and is now being adopted worldwide. While OH presents itself as bridging human and animal health, the majority of advocates and actors taking on the idea can be located in the veterinary sciences. The veterinary origins of the agenda has provoked criticism from some doctors, who perceive OH to be a threat to their professional boundaries: this may account for the limited uptake of OH in mainstream medicine. At the same time, some veterinarians have expressed concern that OH will lead to a loss of the specific status and interest in animal health for its own sake.^25^ This defensiveness over disciplinary boundaries, alongside competitiveness over professional status between veterinarians and their more dominant, better-resourced neighbours, would have been familiar to Schwabe and has repeatedly surfaced in veterinary–medical interactions since the nineteenth century.^26^

To gain a more nuanced understanding of how the ideas associated with OH came about and came together, the specific contexts where the agenda was first developed bear more detailed examination. To this end, I will now explore the longstanding interests and activities of four interlinked advocacy and research networks that were central to the formation of the OH movement: that of Calvin Schwabe, his students and collaborators; the Swiss Tropical and Public Health Institute (STPH); the Wildlife Conservation Society (WCS ); and the One Health Initiative and Commission. Exploring these networks in detail offers further insights into how ideas about OH have moved around and built momentum. By examining the publications, activities, locations and working practices of these groups, we can also understand better the roles they awarded to animals within OH, and how they understood the health relationships between humans and animals.

###  Calvin Schwabe and One Medicine

As we saw in Chapter 10.1007/978-3-319-64337-3_5, Calvin Schwabe initially undertook undergraduate and postgraduate training in zoology, before gaining a veterinary qualification (doctor of veterinary medicine) in 1954, then moving on immediately to retrain in tropical public health, and launching a successful research career in parasitology and epidemiology. He explained in his address to the AVEPM in 2002 that this unorthodox career progression was borne of his desire to combine veterinary practice and scientific research, and ‘to help people in need within poorly “developed” areas of the world’.^27^ It was in 1964, while undertaking consultancy work for the WHO Communicable Diseases Programme and researching the parasitic tapeworm *E. granulosus* at the AUB, that he published the first edition of his most famous work, *Veterinary Medicine and Human Health* (*VMHH*). A combination of textbook and magnum opus, *VMHH* was a product of an age in which (as described in Chapter 10.1007/978-3-319-64337-3_4) interactions between veterinarians and public health experts were increasing. It was intended to advance Schwabe’s view that ‘the veterinarian possesses unique qualifications which can not only be increasingly directed to the investigation of human diseases, but also to their management’.^28^ Written with the support of a Fulbright fellowship and grant from the WHO, the book did not use the term OM. However, its structuring around the well-established domains of public health, epidemiology and comparative medicine, with additional sections on food hygiene and research methods, foregrounded those areas of medicine where animals frequently brought vets and doctors together. The book was reviewed widely in veterinary and medical journals on both sides of the Atlantic (including at least three times by Schwabe’s friend and collaborator James Steele), and it was republished in a second edition in 1969. Later in his career, with the support of a Rockefeller Foundation writing retreat,^29^ Schwabe revised, updated and extended *VMHH* into a third edition, published in 1984. It was here that OM first featured—as a central organizing framework for the volume, in several chapter headings, and throughout the text.

**Fig. 6.2:**
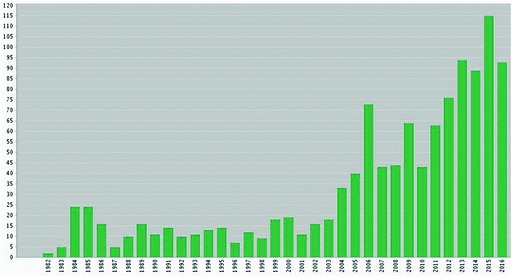
Citations to Schwabe’s *VMHH*, 1964, 1969, 1984. *Source* Web of Science, searched January 2017

As shown in Fig. 6.2, a citation search for *VMHH* suggests that its impact at the time of publication was relatively limited, at least on research publications, and it was not until the mid-2000s that the book was widely cited. The third (1984) edition accounts for about half of the post-2000 citations of *VMHH* and is often referred to in support of the idea that Schwabe devised the concept of OM.^30^ However, searching bibliographic databases reveals that in fact Schwabe was not the first person to use the term OM in the context of human and animal health.^31^ The earliest reference it identifies is an editorial by the physiologist Carl F. Schmidt published in the journal *Circulation Research* in 1962, which extolled the benefits of the OM approach, particularly in the context of space medicine.^32^ Schmidt, a professor of pharmacology at the University of Pennsylvania, cited traditions of veterinary–medical collaboration at the institution going back to the early nineteenth century. Following Schmidt, a series of other references discussing OM were published, mostly by authors associated with the University of Pennsylvania.^33^ Today the leaders of the University of Pennsylvania School of Veterinary Medicine proudly cite these traditions and act as key advocates of OH.^34^ The term had some currency beyond this context, as shown by its appearance as the title of an editorial in the UK’s premier veterinary journal, *Veterinary Record*, in 1975.^35^ Curiously, none of these authors (including Schwabe in the 1984 edition of *VMHH*) provided a definition of OM, instead treating it as a self-evident term which would already be familiar to the reader. This colloquial usage suggests that OM may have arisen organically, perhaps in veterinary-medical teaching or clinical collaborations taking place in and around Pennsylvania, and that rather than inventing it—as claimed by some OH advocates^36^— Schwabe adopted the term and greatly elaborated the idea when working on the third edition of his book.^37^ Further support for this idea comes from following the career of Lord Lawson Soulsby, a recently deceased British veterinarian who served as president of both the Royal College of Veterinary Surgeons and the Royal Society of Medicine. Soulsby, now also claimed posthumously as a ‘One Health pioneer’ and like Schwabe a parasitologist specializing in helminths, held a position as professor of parasitology and chair of the Department of Pathobiology in the University of Pennsylvania School of Veterinary Medicine between 1964 and 1978. He then returned to Britain to head the University of Cambridge Veterinary School. Soulsby was a lifelong practitioner and advocate of integrating human and animal health. It therefore seems plausible that he also picked up the term at Pennsylvania, and﻿ influenced the *Veterinary Record*’s consistent support of OM prior to the rise of OH.^38^

As well as treating OM as a self-evident concept, many of these early authors attributed its origins to their own historical forebears—medical men such as Rudolf Virchow, William Osler and Benjamin Rush, alongside veterinarians such as John McFadyean—whose work transcended the human–animal divide and brought benefits to both. Schwabe went a step further by tracing the origins of OM back to medical and agricultural practices in classical and even prehistoric societies. Twenty-first-century OH advocates have continued this pattern of grounding and legitimizing their work by citing that of leading historical figures, often citing Schwabe’s own historical research as a supporting reference.^39^ Such processes of retrospective citation and celebration of historical individuals and publications are well understood as an important aspect of discipline-building.^40^ However, for scholarly historians, the teleological, progressive historical narratives that they generate are deeply problematic. In attributing the pursuit of a twenty-first-century agenda to intelligent, successful and forward-looking nineteenth- and twentieth-century scientists, advocates for OM and OH have failed to engage with what, in the language of the time, these individuals thought they were doing and why. They have also neglected to consider the specific historical circumstances that encouraged the coming together of human and animal medicine in different times and places, as we describe in this volume and elsewhere.^41^

Despite the widespread recent citation of *VMHH*, the book has been out of print for many years, even if it is widely retained in the libraries of veterinary schools. Its increasing citation rate correlates closely with the increasing usage of the term OH in academic journal articles.^42^ This suggests that it may have taken on some of the features of a ‘citation classic’—a piece written in the past which is widely referenced by contemporary scholars for symbolic or strategic purposes—in this case OH advocacy.^43^ Beyond the book, the figure of Schwabe appears to have been co-opted into this process, playing the role of the latest in a long series of visionary ‘founding fathers’ who each played their part in the foundation of today’s OH agenda. This symbolic role obscures the historical specificities of Schwabe’s own life and work, which, as shown in Chapter 10.1007/978-3-319-64337-3_5, ranged widely across parasitology, epidemiology , anthropology, and human and veterinary medicine. His integrated thinking around health and medicine persisted in his retirement, when he continued his studies on the religious symbolism of cattle in Ancient Egypt, and animal medicine in prehistory.^44^ From his writings on veterinary epidemiology through to the cookbook on utilizing unusual sources of animal protein (*Unmentionable Cuisine*), most of Schwabe’s work supported his continually restated argument that veterinary medicine was important for human public health, and that there should be a legitimate space for veterinarians to contribute to that goal.^45^

During his lifetime, Schwabe influenced wider thinking on health and medicine, primarily via his immediate, largely veterinary, network of collaborators and students, and in turn their collaborators and students. As exemplified by the contributors to the 2002 Schwabe symposium, over time, members of this network moved into positions of power and influence both within US health policy and across international health. Two years after the symposium, the contributing papers were published in a special issue of the journal *Preventative Veterinary Medicine*.^46^ While we do not have access to the attendee list, the stories presented below suggest that this event may have had a catalysing influence on the subsequent construction and promotion of the OH agenda in several institutions.

### The Wildlife Conservation Society

The Wildlife Conservation Society (WCS) constitutes a very different working context from that experienced by Schwabe, yet one in which his ideas about human and animal health helped to provide a foundation from which new interconnections across these domains developed. The WCS is a USA-based non-governmental organization. Today it describes its core vision as ‘a world where wildlife thrives in healthy lands and seas, valued by societies that embrace and benefit from the diversity and integrity of life on earth’.^47^ It can be regarded as a US equivalent to the Zoological Society of London, and it runs several zoos and wildlife parks in the USA, including the Bronx Zoo. In addition the WCS runs an international programme of field research and conservation projects; fundraising, policy-making and campaigning; and it has pioneered veterinary wildlife research and practice in its zoos and in the field.

From the late 1990s, a small group of﻿ veterinarians working﻿ at the WCS (including William Karesh and Steven Osofsky) started building collaborative links between themselves, scientists studying emerging infectious diseases, and veterinary scientists and clinicians involved with global livestock health.^48^ In 2003 they co-organized a workshop in Durban, South Africa, with the Veterinary Specialist Group of the International Union for the Conservation of Nature ﻿(IUCN). The purpose of the meeting was to further this collaborative agenda, bringing in practitioners working in government, international health and conservation non-governmental organizations to launch a new international network for health, environment and development, entitled AHEAD. In the workshop briefing, the organizers laid out an agenda for developing better collaborative relationships across these fields, which they described as “One Health” .^49^ The following year the WCS group organized an international conference on the theme of One World, One Health (OWOH), which was sponsored by the Rockefeller Foundation and held at the foundation’s university campus in New York. Over the next few years it built links with the international Food and Agriculture Organisation (FAO) and the World Organisation for Animal Health (OIE), contributed to international responses to HPAI, and continued to publish its ideas about OH in academic and policy journals.^50^ The proceedings of the AHEAD workshop outlined the WCS group’s﻿ ideas about OH in more detail. Starting with the WHO’s widely accepted definition of ‘health’ as a state of positive wellbeing (rather than the absence of disease), advocates of ‘ecosystem health’ argue by analogy that ecosystems can also be regarded as patients and evaluated as healthy or otherwise. The WCS veterinarians built on this idea, bringing it together with Schwabe’s ‘visionary attempts … to construct a bridge between medicine and agriculture’ to argue for a broader collaborative approach to improving the health of humans, animals and environments, which they described as OH.^51^

These wildlife veterinarians developed the idea of OH primarily to advance their conservation agenda, but it was particularly grounded in thinking about problems of infectious disease, especially zoonoses. Their thinking around zoonotic diseases was, and is, unusually broad. While the WHO defines ‘zoonosis’ as disease transmission between animals and humans, most actors in global public health today use the term to denote the transmission of infections *from* animals *to* humans. By contrast the WCS group ﻿and their collaborators discuss the movement of infectious diseases between multiple species, back and forth across wildlife and livestock, humans and animals, much as Schwabe did when challenging the human–animal distinction at the core of ‘zoonosis’.^52^ If anything, the WCS group took this even further, highlighting the particular risks of transmitting common human infectious diseases *to* endangered species of wildlife. This understanding—that humans form part of an interconnected network of organisms including wildlife, domestic animals and microorganisms—was underlined by the logo the group used for OWOH, which depicted a ‘parade’ of silhouetted wild and domestic animal species alongside a human adult and child.^53^ In the WCS group’s writing on OH, animals commonly appear as specific species rooted in specific places, such as buffalo suffering from rinderpest in Uganda; or gorillas, bushmeat and Ebola in the Congo. While authors award animals key roles as transmitters of infectious diseases, they also present them as charismatic wildlife that need to be protected from disease; or, in the case of livestock animals, as potential food for humans whose productivity (like that of the cows discussed in Chapter 10.1007/978-3-319-64337-3_4) must be safeguarded and promoted. While the WCS veterinarians’ ultimate priority was (in line with their institutional orientation) the protection and preservation of wildlife, the version of OH that they fashioned argues that this is best pursued through means that jointly protect human, animal and ecological health, for the benefit of all species. While drawing heavily on older ideas about OM, by broadening the scope from medicine to health, and bringing in the idea of care for ecosystems and wildlife alongside livestock and humans, it decentred humans significantly.

### The Swiss Tropical and Public Health Institute

Drawing on these developments, in 2005, Professor Jakob Zinsstag and a group of his﻿ colleagues at the Swiss Tropical Public Health Institute (STPH) published an article in the international medical journal *The Lancet* arguing for the OH approach.^54^ Bringing OH to a much bigger audience, including medical doctors and public health professionals, Zinsstag et al. started their article with a discussion of Schwabe and OM (citing *VMHH*) before proceeding to discuss the importance of ecosystem health (citing the WCS). They argued that OH needed to extend beyond the specifics of human and veterinary medicine and include broader ideas about health as wellbeing. They, in turn, added their own perspectives, to place a greater emphasis on research into tropical medicine and livestock health. They also introduced the public health concept of ‘health systems’, defined by the WHO as ‘all organizations, people and actions whose primary intent is to promote, restore or maintain health. This includes efforts to influence determinants of health as well as more direct health-improving activities’.^55^ This further broadened OH by highlighting the social and administrative aspects of healthcare. Zinsstag and his colleagues applied this idea to human and animal health by arguing that, for example, vaccinating animals against diseases such as brucellosis or rabies can simultaneously protect human populations.^56^

The STPH is a partly state-funded research institute, devoted to research and clinical practice in tropical diseases and global public health. It was founded in 1943 by the zoologist Rudolf Geigy, who directed the institute until 1975.^57^ The original aim of the then Swiss Tropical Institute was twofold: to perform research into tropical diseases—a field that straddled human medicine, biology and agriculture from its very foundation, as explained in Chapter 10.1007/978-3-319-64337-3_5 58—and to train scientists, administrators and others preparing to live and work in French and British colonies.^59^ Geigy himself worked across multiple disciplines, including zoology, physiology and embryology, and the institute was organized along these lines. From early in its history, the STPH worked with an affiliated research institute in Cote D’Ivoire, the Centre Suisse de Recherches Scientifiques (CSRS), to enable longstanding collaborative partnerships between Swiss and Ivoirian scientists investigating tropical medicine.^60^ Today it is a large and thriving organization, with divisions focused on epidemiology , parasitology, international health, diagnostics, drug development and education.

Working in this tradition, Jakob Zinsstag joined the STPH during the 1990s to perform postdoctoral research on trypanosomiasis in the Gambia. After spending four years directing the CSRS in Cote D’Ivoire, he then returned to Switzerland to head up the STPH’s animal health research group. For many years this group has worked on topics in and around global livestock health and international development, including the epidemiology of zoonotic and parasitic diseases , and also public health interventions such as vaccination, as described above. As suggested by the STPH’s institutional orientation towards tropical medicine, the group’s work is mostly conducted in the global South, with a particular focus on working with pastoralist communities. It is clear from the STPH group’s publications that—like the WCS veterinarians—they were working across human and animal health long before it started using the term OH in the mid-2000s.^61^

Alongside their emphasis on health systems, the STPH group also advocate a ‘transdisciplinary’ approach to OH research and practice, which involves working in participatory partnerships with local communities.^62^ In contrast to the WCS group, who work across science and policy from within a non-governmental organization, the engagement of Zinsstag and his colleagues with OH has involved primarily academic activities, such as organizing professional societies, attending conferences, and writing and editing research articles. When they write about OH, the STPH group move rapidly beyond generic discussions of humans and animals towards the specific: it works with particular kinds of people and animals (e.g. mothers, herders, cattle, dogs ) on particular diseases (e.g. rabies, brucellosis, Q fever) and in specific places (e.g. Chad, Cote d’Ivoire, Morocco). Highlighting how animals and humans live together in communities or as part of ecosystems, they consider it essential to care for the health of all. While the WCS group sometimes refer to wild animals, usually as disease vectors, the core of their work is with domestic animals, which are awarded roles as food sources, working animals, companions and community members.^63^ As STPH’s overall focus on human public health implies, this group’s version of human–animal health is, like Schwabe’s, anthropocentric, albeit with an intense interest in the shared lives and wellbeing of humans and animals, the transmission of infections between the two and the ambition to protect both.

### The One Health Initiative and Commission

In 2006, the year after the STPH group’s *Lancet* article, the incoming president of the American Veterinary Medical Association (AVMA), Dr Roger Mahr, addressed the association’s annual conference. He argued that twenty-first-century challenges of global food security and zoonotic diseases meant that ‘the continuing convergence of animal health, human health, and ecosystem health is the new reality’, which must be responded to with a ‘One World, One Health, One Medicine’ approach. Mahr argued that veterinarians should adopt OH. Through building partnerships with public and environmental health professionals, they should assume leading roles in meeting these challenges—and growing the profession along the way.^64^ Over the following year, he gained the support of his counterpart at the American Medical Association (AMA), public health physician Ronald M. Davis, who had pre-existing concerns about the risks that zoonotic diseases pose to humans. While this alliance resulted in OH being endorsed by the AMA in 2007, Davis died of cancer the following year and the resolution was subsequently dropped, indicating that the US medical profession more widely did not share his enthusiasm for OH.^65^

Mahr was more successful in persuading his colleagues in the AVMA, who passed their own resolution supporting OH. They also established a One Health Commission to investigate ways of improving veterinary-medical collaborations and moving the agenda forward, chaired by Lonnie King, one of the 2002 Schwabe symposium contributors. In parallel, Laura Kahn, Thomas Monath and Bruce Kaplan formed the One Health Initiative, an unfunded advocacy group dedicated to making the case for OH. Kahn is a physician based at Princeton University who by 2006 was already working on biosecurity and the risks of pandemic disease.^66^ Kaplan had worked on food-borne illnesses for the Centres for Disease Control and Prevention (CDC) before his retirement, and Monath is a physician and consultant working in the pharmaceutical industry.^67^ The OH Initiative quickly launched a website and organized a series of meetings and publications on OH/OM, including a piece entitled ‘Confronting Zoonoses’ co-authored by Kahn and Kaplan with veterinary public health pioneer James H. Steele (whose role in the 1948 establishment of the WHO’s Veterinary Public Health unit is described in Chapter 10.1007/978-3-319-64337-3_4.)^68^ When the OH Commission published its report in 2008, it acknowledged the central influence of Schwabe’s *VMHH*, cited Zinsstag et al.’s *Lancet* paper, the literature on emerging infectious diseases and the OH Initiative’s publications. However, it did not cite the WCS veterinarians, instead tracing their influences directly back to Schwabe and OM—a move which emphasized veterinary medicine and de-emphasized environments and wildlife.^69^

The differences between the OH Initiative and WCS versions of OH are reflected in the imagery used by each group: unlike the WCS’s ‘parade’ of humans and other animals, the OH Initiative’s logo depicts the twinned icons of human and veterinary medicine in front of planet Earth.^70^ This signals that for the Initiative—as for the Commission—OH is a project for changing professional relationships that is grounded in and legitimized by human–animal health relationships. Its assumption that closer veterinary–medical partnerships are universally relevant and beneficial perhaps explains the somewhat generic status of many of the animals that feature in their writings. The categories of animals, animal health, animal disease and animal science feature much more frequently than specific instances, and diseases (e.g. bovine spongiform encephalopathy—BSE) are named as or more often than the animals involved (e.g. cows). The key roles awarded to animals are those of patients; transmitters of zoonotic infections *from* animals *to* humans; victims of environmental pollution; and models and subjects of biomedical research.^71^ The animal whose health has been of central concern to these groups has tended to be the human animal, with a particular focus on the USA. Like the immediate network around Calvin Schwabe, the OH Initiative and OH Commission maintain close connections with US health policy organizations such as the CDC and the Food and Drug Administration.^72^

In October 2008 a group of international agencies, including the WHO, FAO, OIE and World Bank, published a ‘Strategic Framework for Reducing Risks of Infectious Diseases at the Animal–Human– Ecosystems Interface.’ This 70-page document drew upon the OH Commission report alongside the work of the WCS group to reflect on problems encountered during the avian influenza outbreak, when the division of health between animal- and human-focused organizations prevented effective communication and the ability to quickly coordinate responses to this ‘hybrid’ disease.^73^ The agencies put forward proposals to improve the situation, using OH as an organizing framework and as shorthand to signal their collaborative intent.^74^ In 2010 they followed up with a shorter policy briefing that reinforced their commitment to OH.^75^ These publications brought the ideas and terminology of OH to the attention of a wider range of audiences than ever before. After 2010, OH became much more coherent and continued to grow, with increases in academic citations, the establishment of academic journals, textbook publications, the adoption of the term in health policy in several countries, and its uptake by major funders of global health agencies, including the Gates Foundation.

This pattern—of slow emergence, usefully flexible terms, intense negotiation followed by broad consensus, and then widespread adoption—was first described as a ‘scientific bandwagon’in studies of the emergence and spread of molecular biology into cancer research during the 1980s.^76^ However, while OH broadly fits this description, there are some key differences. In particular, it works across multiple disciplines and beyond science into the policy sphere; it is aimed at large institutional as well as individual actors; and it employs ‘buzzwords’ which are not only usefully flexible but also help advocates gather rhetorical support and financial resources from supporters. OH can therefore be viewed as an example of a newer form of scientific agenda-building, the ‘ interdisciplinary bandwagon’, which works in concert with other agendas such as food security and translational medicine.^77^

While the networks associated with Schwabe, the WCS, the STPH and the OH Initiative have by no means been the only groups involved in building OH, they can be understood as core to the early negotiation and development of its agenda. By looking at their writing and activities, we can identify the range of different influences, institutions and intentions that have given rise to not one but many OHs. We can also see that while the widespread belief that Schwabe founded OH/OM is inaccurate, his ideas, as propagated by his collaborators, students and readers, did exert a profound influence on the early formation of OH. In this sense, Schwabe was indeed crucial to the development of OH. However, the rise of OH also drove the popularity and fame of Schwabe, particularly after his death in 2006. It is also important to note that, in the majority of cases, the OH advocates discussed here were *already working and thinking across human and animal health* before OH rose to prominence. For them, Schwabe and the OH concept provided a concise and compelling way of articulating the advantages of working in this way—to themselves, to colleagues, to researchers beyond their immediate fields, and to wider audiences including policy-makers and funding bodies. In turn, each of these groups has been successful in persuading and enrolling each other, alongside an increasingly wide circle of individuals and organizations, into their common cause.

Despite these successes, it would appear that the consensus around what OH is, and, perhaps more importantly, what it should be, is still quite fragile. As we have seen, while the various groups described above have been able to develop and adapt each other’s ideas, recrafting OH to fit their own contexts while also working together, multiple versions of OH remain in play. In particular, the two sides of the OH umbrella (Fig. 6.1) align not only with different fields of interest but also with different versions of human– animal relations. OWOH, the slogan originally used by the WCS group, can be broadly characterized as the left-hand side of the umbrella, involving veterinarians but also conservation, global health and development specialists. As we have seen with both the WCS and the STPH groups, OWOH sees humans, animals and environments as part of an interconnected network which requires care, decentring humans partially or completely. In contrast, the twenty-first-century version of OH/OM broadly but not completely aligns with the right-hand side of the umbrella: in something of a departure from Schwabe’s original ideas it mobilizes zoonoses as a concern primarily because of the risk posed to humans. It then interconnects into clinical practice and human medicine via translational medicine, where it highlights the benefits of veterinary–medical collaboration for developing new drugs, gaining financial and symbolic support from pharmaceutical companies.^78^ The emphasis on human health risks alongside veterinary–medical professional relationships creates a version of OH which appears to be significantly more anthropocentric than that seen in OWOH.

While there is no direct evidence of active tensions between OWOH and OM, the OH consensus does appear to be fracturing somewhat. For example, the WCS group have reoriented themselves towards ‘ ecohealth’—a restatement of the ideas of ecosystem health, with key personnel such as William Karesh taking on leadership roles in a new Ecohealth Alliance.^79^ Jakob Zinsstag and his colleagues have also taken on core roles in the international academic association for EcoHealth. Other researchers working across human and animal health are adopting newer buzzwords alongside or instead of OH. A good example of this is ‘the nexus’,^80^ an idea that originated in environmental governance during the late 2000s and that describes the need for interlinked thinking in response to environmental challenges that cut across multiple domains. Unlike OH, it has avoided defining what these domains are, and this increased flexibility may be helping ‘the nexus’ to avoid becoming as entangled in disciplinary politics.^81^

## The Animal Subjects of One Health

While the above stories bring depth to our account of the rise of OH in the early twenty-first century, so far this chapter has told a mostly human story, albeit one involving several groups of people professionally involved with animals. In keeping with the overall aims of this book, I shall now analyse the place of animals within OH, both in their own right and in their relationships with humans. Looking at how animals are conceptualized, represented and acted on by those who research, practice and advocate OH offers important insights into the roles awarded to these animals and their relative valuation in society, health and medicine. In revealing how humans perceive and have elected to respond to the health threats and opportunities presented by animal bodies, it also illuminates the ways in which animals have inadvertently shaped OH, and how OH aspires to shape them. These insights derive from analysis of the literal and symbolic traces that animals have left on the scientific journal articles, websites, policy documents and media outputs created by human participants in OH.

### Animals in One Health Research Texts

**Fig. 6.3:**
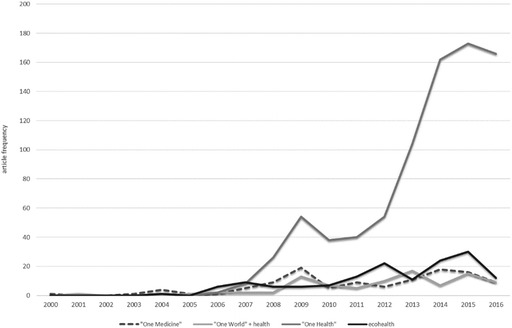
Growth of the One Health bandwagon. *Source* Web of Science, searched March 2017

While OH encompasses research, policy and clinical practice, the preceding stories show that scientists and their research lie at its core. Consequently, scientific citation databases can reveal its entry into, and expression in, research agendas, as shown in the earlier analysis of Schwabe’s *VMHH*. The development of the OH bandwagon can be traced in the same way: usage of the term in journal articles spiked after the WHO-FAO-OIE joint statement, and increased sharply between 2012 and 2016 (Fig. 6.3).^82^ Analysis of these references by subject area throws further doubt on the ambitions of OH advocates to work across and/or beyond the boundaries of human, animal and environmental health. More than 60% of publications discussing OH are published in veterinary science journals, with a limited reach into fields of human medicine, such as infectious diseases and public health, and very few citations in other biomedical or environmental science journals.^83^ This is supported by a recent analysis of the literature on dynamic disease modelling (a technique used in veterinary, medical and ecological sciences), which found three distinct publication ‘silos’: one in ecology, one in veterinary medicine and a third multidisciplinary group dominated by epidemiology, statistics and public health. Between 1990 and 2015, the three groups remained distinct, maintaining different methodological practices, and while ecologists and veterinarians increasingly cited authors from the third group, they did not cite each other.^84^

The capacity to search by keyword also provides a direct technique for ‘following the animals’ in OH research, telling us which animals actually feature in journal publications and offering some insight into what roles they are awarded by researchers. The Web of Science database was therefore searched for ‘ one health’, alongside a series of animal names and roles. The relative frequency of these terms and the scientific content of the articles in which they feature were recorded. The results of this process are shown in Tables 6.1 and 6.2. Perhaps the most striking finding is that the most frequently mentioned animal in the scientific literature on OH is actually the *human* animal, while non-human animals are referred to mostly in generic terms. The roles that animals were awarded in scientific research are indicated via the use of categories such as ‘models’ (model organisms employed as experimental material in human biomedical research), ‘food’ (as a source of human infection, rather than nutrition, as discussed in Chapter 10.1007/978-3-319-64337-3_4), ‘wildlife’ (hosts and transmitters of zoonotic infections), ‘livestock’ (intermediate hosts and transmitters of parasites, as described in Chapter 10.1007/978-3-319-64337-3_5, and as zoonotic disease transmitters), and ‘ pets/companion animals’ (again as zoonotic disease transmitters). Many of the journal articles using these terms demonstrate a lack of specificity about what aspects of health or disease are actually of concern.

**Table 6.1:** One Health and animal categories

*Search term*	*Article hits*
‘ One Health’	1737
OH AND human	1178
OH AND animal OR animals	727
OH AND model	293
OH AND food	170
OH AND wildlife OR ‘wild animal’	125
OH AND livestock	115
OH AND pet OR companion	85

*Source* Web of Science, 2004–2015, searched January 2017

**Table 6.2:** One Health and animal species

*Search term*	*Article hits*	*Research topics*
OH AND canine OR dogs	121	zoonosis (46), rabies (38), vector (31), bite (22)
OH AND avian OR poultry OR birds	94	influenza (66), zoonosis (42), food (28)
OH AND bovine OR cows OR cattle	80	zoonosis (34), TB (25), food /milk (24), brucellosis (11),
OH AND swine OR pig	57	zoonosis (27), influenza (16), model (15), food (15)
OH AND feline OR cat	39	zoonosis (15), parasite (10),
OH AND horse OR equine	25	zoonosis (11), hendra (6)
OH AND rat OR mouse OR rodents	18	model (11), zoonosis (6), leptospirosis (3)
OH AND sheep OR goat	18	zoonosis (6), rift valley fever (3); brucellosis (2)
OH AND bat	17	rabies (7), zoonosis (6), hendra (5)
OH AND deer	12	TB (5), zoonosis (2)
OH AND gorilla OR chimpanzee OR ape OR monkeys	10	conservation (5), zoonosis (4)

*Source* Web of Science, 2004–2015

Far fewer OH articles mention specific types of animal, and when they do, a few species dominate (Table 6.2). This makes it possible to examine the articles more closely and analyse the specific animal contributions to human health that researchers are interested in. Dogs feature most commonly, primarily as vectors of zoonotic disease, particularly rabies, and as direct threats to human health via bites (which also carry risks of disease transmission); then birds, primarily as vectors of influenza, but domestic poultry specifically feature as a major source of gastrointestinal infections, such as *Salmonella*. Cows are the third most common animal type, featuring usually in relation to zoonotic infections carried in milk and meat, such as bovine tuberculosis and brucellosis.^85^ A second key animal role that emerges is that of the experimental model for human disease, and it is usually assigned to rodents or pigs, reflecting the intersection between OH and translational medicine.^86^ Despite the prominence of the category ‘wildlife’, specific species are rarely named. When they are it is in relation to certain infections, such as rats and leptospirosis, or bats and viral infections such as rabies or Hendra.^87^

From these figures it appears that much of the research literature using the term OH tends to discuss animals in terms of generic categories (e.g. animal-livestock-wildlife). When specific types of animal are named, they tend to be domestic species which pose certain risks (as disease vectors, e.g . dogs-rabies, birds-influenza , cows- tuberculosis) or offer benefits (as experimental models, e.g. rodents ) to humans. This strong focus on the animal roles of disease vector and experimental model represents the continuation of existing interests in their relationships to human health that date back to the nineteenth century.^88^ It supports the idea that OH can be understood in part as a rebranding of existing fields of enquiry. While OH advocates may claim to pursue an expansive vision of health at the interface of humans, animals and environment, in practice OH is primarily shaped by pre-existing, longstanding human–animal health relationships. This analysis suggests that the majority of researchers adhere to an anthropocentric perspective in which animals matter only insofar as their bodies threaten human health or offer opportunities for its advancement.^89^ Animals are thereby sidelined as prospective beneficiaries of OH. Despite this, it is worth noting that searches of Web of Science for these animal types alongside terms such as ‘zoonosis’ reveals an abundance of scientific research primarily concerned with animal health and its relationships with humans and/or environments. However, for reasons which merit further investigation, the individuals who conduct this research do not appear to find OH a useful term for advancing their ideas.

###  Animals in One Health Imagery

Animal images are central to the visual strategies employed by OH advocates as they seek to persuade colleagues, funders and policy-makers of OH’s merits. These images appear occasionally in journal articles, but more prominently in the ‘grey’ literature of policy reports, the websites of conference and advocacy groups, and mass media coverage of OH. This material can offer further insights, beyond the more constrained form of scientific publishing, into the place of animals in OH. Its imagery consists of logos, infographics, diagrams and photographs, which are used in a variety of ways: most obviously to envisage ideal relationships between human, animal and environmental health; as logos highlighting the ‘brand’ of particular organizations and events; and to illustrate specific examples of the OH approach. Animals and ideas about animals appear throughout, alongside more ‘realistic’ photographic images of people, and of human–animal interactions.

Much of this material is available online and can therefore be collected easily via keyword searching. A sample of OH images was collected using two main routes: by searching on Google for images relating to ‘ one health’, ‘ one medicine’ and ‘one world, one health’; and by harvesting images directly from OH advocacy websites and policy documents. This activity was performed initially in 2013 and then repeated in late 2015, creating a total sample of 217 image files. Of these images, approximately 60% were drawn illustrations, infographics or logos, while the rest comprised photographic images. Analysis of the roles and relationships they award to animals indicates some commonalities and also some significant contrasts with what we have seen so far.

**Fig. 6.4:**
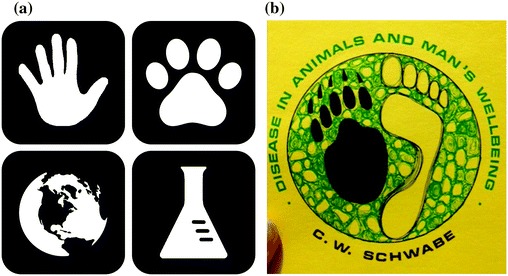
Human–animal partnerships.* Sources* a) Cornell One Health workshop, 2015; b) Calvin Schwabe, *Disease in Animals and Man’s Wellbeing* (1970)

Given that the core idea of OH involves intersecting domains, the vast majority of these images provide visual interpretations of this concept, depicting the human– animal dyad of OM, a triad of humans, animals and the environment and so on. The images used to convey these broad, abstract categories have been, on the whole, correspondingly broad and abstract. Indeed, the most abstract of these dispense with any form of direct representation, opting instead for interconnected spirals or swirls.^90^ The logo of the OH Initiative (as described above) depicts the paired icons of human and veterinary medicine. A minority of images in the sample use this same idea. An alternate approach depicts the subjects of OH rather than the professions concerned with it. Several versions of this strategy can be seen in the sample. The simplest and most popular version uses the iconography of a human hand or foot alongside animal feet, most commonly a paw (Fig. 6.4).

While the ‘human’ in these images is universal, the ‘animal’, while deeply abstracted, is not. The animals with paws indicated in Fig. 6.3 tend to be carnivores: in veterinary contexts, paws often indicate pet dogs and cats. This inference is backed up by the photographic images in the sample, which includes eight pictures of dogs, all depicted as companion animals and sometimes as veterinary patients (one cat features, accompanied by a dog). By contrast, in the scientific articles analysed above, these roles rarely feature. There, dogs appear primarily as risks to humans either via bites or as a source of rabies infection.

**Fig. 6.5:**
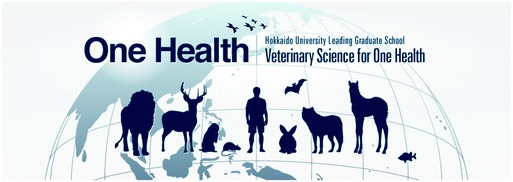
OH contextualised.* Source* One Health Graduate School, Hokkaido University, Japan

The transition from OM to OH involved a shift from a clinical, medical focus to broader concepts of health and wellbeing. It also extended beyond human–animal health relationships to include the idea of health across the natural environment. This shift is reflected in the usage of tripartite imagery depicting humans, animals and environments, which was first employed by the WCS vets in their 2003 AHEAD workshop.^91^ In these logos, the hands and paws are joined by leaves, trees or sheaves of wheat. Alternatively, humans, animals and plants are depicted as silhouettes, and this strategy introduces a little more variety into the imagery. Humans are joined by smaller figures evoking families, and, while paws still feature, livestock animals such as cows or pigs also appear.^92^ Human and animal silhouettes are also used in an alternative strategy to depict humans–animals–environment, bringing together the ‘parade’ used by the 2004 OWOH meeting with the planetary imagery of the OH Initiative. A good example of this type can be seen in the logo designed by Hokkaido University (Fig. 6.5). Humans and animals are depicted together, in or on the planet Earth. While most of these images remain universal and generic, others like this one display a diversity of domestic and wild animals, which are used to convey a particular place: the more generic lion, deer, horse and others are joined by the locally specific Japanese macaque and the extinct Hokkaido wolf. Images of planet Earth, circular imagery, and/or of the planet held in human hands featured in 35 images from the sample. It seems plausible that these logos draw on global health and environmental campaigning, where the ‘globe in hands’ motif is also used widely, deriving as it does from the iconic 1972 Apollo ‘ Blue Marble’ portrait of the Earth.^93^

More ‘realistic’ images and photographs are employed in two key ways: as a less design-oriented version of the OH ‘tripartite’ of humans-animals- environment, or to illustrate examples of problems or research issues which OH can address (e.g. influenza illustrated by chickens). The animals portrayed in these realistic tripartites are usually domesticated—both agricultural animals and pets, although more frequently the former—while images of plants (representing the environment) tend to suggest food crops.^94^ Photographic images are also employed to illustrate examples of problems that can be addressed through the OH approach. Conveying ‘success stories’ in this way is so important that one OH advocacy site hosts an online archive of images specifically for promotional usage.^95^ Almost all of these types of image include animals, but they are dominated by very particular types of animal, playing particular roles in particular places. For example, of the 11 photographs of cows in our sample, 7 are of zebu-type cattle, often depicted with African pastoralist farmers. As we have seen in Chapter 10.1007/978-3-319-64337-3_4, the zebu was a core animal of concern for veterinarians involved in international health and nutrition in the mid-twentieth century, whose work Schwabe was closely associated with. Their visual presence suggests that pastoralist contexts remain a key site for OH in the present day. Chickens and pigs also feature. Their presence reflects OH’s entanglement with contemporary concerns about pandemic influenza, and resonates with the content of the scientific journal literature, as analysed above. These species are shown with humans in small-scale, global South farming contexts; being transported or contained for sale in ‘wet markets’ in East Asia; or (in the case of chickens) with humans in medical protective clothing. Here, animals and humans are portrayed in close proximity, in open air and/or muddy, messy situations, implying that the source of zoonotic disease risks are not solely the animals but the intimacy of human– animal relationships in these parts of the world.

Wild animals appear in some of these photographs, usually large charismatic species, including ostriches, giraffes, seals, elephants, lions and great apes. They are depicted alone, or in about a third of the images with humans, usually as recipients of veterinary care, but occasionally as ‘hands and paws’, evoking the imagery seen in Fig. 6.5, and the famous *National Geographic* image of Jane Goodall and a chimpanzee hand in hand. These images seem to be drawing on conservation and animal welfare campaigning to advance ideas of human care for, and custodianship of, animals.^96^ While occasionally wild animals appear in relation to zoonotic disease transmission, these are usually less sympathetic animals such as bats, at times manipulated to look more threatening.^97^ The prominence of wildlife in these images contrasts heavily with the published literature on OH, where beyond the generic category of ‘wild animals’, individual species barely feature.

Eight photographs in the sample show animals as patients, with human clinicians working directly with them in one way or another. They are split evenly between images of dogs being cared for in clinical settings (by implication in the global North), and wild or agricultural species being cared for in the field (by implication in the global South). Finally, 18 images of humans without animals feature. About half of these are pictures taken at conferences and other scientific or policy meetings, while most of the rest depict people working in laboratories. With the exception of a still from the 2011 film, no images of human patients were found. Indeed, the plot of *Contagion*, which plays out the imagined scenario of an emerging infectious disease pandemic, can be seen as part of a broader cultural case for OH. The film starts with an American businesswoman who becomes sick after a trip to Hong Kong, passing on the disease to several others before dying and setting off a civilization-threatening pandemic. While much of the film focuses on victims, survivors and scientists’ efforts to understand, trace the source of and treat the outbreak, the end of the film provides a critical ‘reveal’. A series of brief shots shows the destruction of rainforest, disrupted bats taking refuge in a pig house, a pig sold at a wet market being prepared in a restaurant, an Asian chef with unwashed hands, and an American businesswoman, who then becomes ill, taking the viewer back to the start of the story. This narrative brings together many of the themes found across OH advocacy images. Indeed, Professor Ian Lipkin of Columbia University (an expert in emerging infectious diseases and OH ally) was a key scientific adviser for the film.^98^

Taken together, what can these images tell us about the sorts of animal bodies and disease that have shaped OH, and which are, in turn, shaped by it? Generic animals, humans and plants play a central role—with outlined images of human figures, common animals such as cows, hand-foot-paw prints, and leaves often appearing in OH logos. Reinforcing the verbal messages of OH advocates, they convey an idea of OH as a generic, universal approach that addresses all types of health problem arising at the intersections of humans, animals and environment—even though, as shown above, the work performed by researchers adopts a more selective approach to those problems. Once we move beyond the generic animal, more differences begin to open up between their portrayal in OH images, advocacy arguments and OH research. For example, while cows feature in a small number of OH research papers which are largely devoted to bovine tuberculosis and brucellosis, they are extremely common in the visual sample. While dogs appear in both, in visual images they feature as family members and veterinary patients, not—as in scientific articles—as carriers of rabies. Images of charismatic wildlife such as great apes and giraffes are prominent in OH advocacy but extremely rare in OH research. By contrast, animals as experimental models are rarely depicted despite their presence in the research literature. These are obvious promotional choices, making the most of publicly appealing imagery while avoiding drawing attention to more controversial animal roles. However, this once again highlights contrasts between OH promotion and practice.

These images can also tell us how OH advocates understand relationships between humans, animals and environments—as they are and how OH believes they should be. Humans appear regularly, talking to each other, caring for animals, working in laboratories, or as human hands. OH depicts itself as a form of care of animals, environments and the planet. Implicitly or explicitly, this imagery shares the assumption that humans must care via forms of custodianship where only human agency is made visible. In first world contexts we see familial or clinical scenes, most often with pets; in images of the global South and East we see small-scale, direct agricultural care and veterinary care of wildlife. In this way, OH indicates its globalizing ambition alongside its awareness of key differences in human–animal relationships in different parts of the world. Care and risk are held in balance, particularly in the context of zoonotic diseases, which by implication come from less appealing wildlife species and from modes of human–animal interactions that occur in traditional food and farming systems. This narrative casts animals and certain kinds of human as ‘guilty victims’, geographically far away from the global North, or socially excluded, who must be kept out at all costs.^99^ It is also at odds with emerging OH research which suggests that pandemic disease risks may be accentuated not by traditional but by modern intensive farming systems and the global distribution of their products.^100^ Finally, it is worth observing that as time goes on, the imagery of OH has become more and more abstracted: following John Berger, the animals are literally disappearing from a movement primarily led by people concerned with animal health.^101^

## Conclusion

This chapter has examined attempts in the early twenty-first century to develop integrated OH approaches to problems lying at the intersection of human health, animal health and the environment. It has explored in detail the early formation and negotiation of OH, teasing out the diverse institutional contexts in which it emerged and the different versions of OH they gave rise to. The roles played by Calvin Schwabe in the development of these many versions of ‘One Health’ have been investigated: while he was not in fact the originator of OH or OM, he has provided a common point of connection and inspiration across the key scientific networks involved in building what has, in very recent years, become an ‘interdisciplinary bandwagon’. Alongside a series of august forebears, Schwabe is now cited as a key precedent, informant and source of authority for OH today. However, as this chapter has demonstrated, the OH agenda developed this role for Schwabe, just as much as he—through his ideas, publications and personal connections—developed the modern movement for OH.

In keeping with the overall aims of this book, this chapter has also indicated the sorts of animal and animal health concern that have attracted attention from across human medicine, veterinary medicine and the life sciences, and contributed to the development of new relationships between individuals working in these fields under the banner of OH. Both scientific journal articles and images in OH advocacy documents deal overwhelmingly with animals in the abstract: as broad categories such as ‘animal’ or ‘ wildlife’, and as literal abstractions in the form of silhouettes and logos. This abstraction matches the generic claims made for OH by its advocates, as a universally applicable and beneficial approach to health that has demonstrated its value repeatedly through history. It also recalls to mind how often and how persistently animals continue to be viewed as ‘mere blank pages onto which humans wrote meaning’.^102^ At the same time, when tracing the origins and practical applications of OH, very specific types of animal, context and problem are shown to be involved.

Zoonotic diseases (as transmitted by agricultural animals , dogs and wildlife) feature﻿ prominently in both the research and policy/advocacy literatures analysed here, while animals as ‘models’ in laboratory research appear much more commonly in the scientific literature. As our annotated bibliography  indicates, both are extremely longstanding animal roles. When considered alongside the institutional origins of OH, their prominence suggests that much of what goes by the name of OH today is in fact a rebranding of existing health agendas. They also indicate an anthropocentric character to OH, its greater concern for the human than the animal health benefits that may arise from an integrated approach to their health. While OH imagery demonstrates an additional objective of care for valued animals (livestock , pets and aesthetically pleasing charismatic wildlife), which it portrays in the roles of patients and subjects of human custodianship, this has yet to be realized substantially in scientific work that claims to pursue a OH approach. This situation indicates the peculiar contradictions at the heart of OH: a movement trying to bring together human and animal health does so by arguing—and working to ensure—that attending to animal health will benefit humans.

These anthropocentric contradictions may be related to ongoing anxieties about the ability of OH to move ‘from rhetoric to reality’.^103^ As both advocates and observers have noted, OH has tended to flip from very broad generalities to specific ‘success stories’, but with little discussion of how researchers, policy-makers and clinicians might move from one to the other.^104^ The analysis presented here suggests that these problems persist at the level of research practice. Yet OH continues to be mobilized in international health. Over the past two years the WHO has published strategies for action on antimicrobial resistance and rabies elimination, which both prominently reference OH as a conceptual support for coordinating across organizations in human and animal health.^105^ Time will tell what impact such activities have on the roles that animals perform in OH, on how OH is practised, and on how it presents itself to the wider world.

## Footnotes

1 At the time the group was known as the Association of Teachers of Veterinary Public Health and Preventive Medicine. The symposium became an annual event, at which a senior member of the field is presented with the Calvin W. Schwabe Award. For details of the original meeting, see AVEPM (2002) p. 3.

2 Schwabe (2004).

3 Schwabe (1984) pp. 1–2.

4 Compare Schwabe (1964, 1969) with Schwabe (1984).

5 See all papers in *Preventive Veterinary Medicine* 62 (2004).

6 Osofsky et al. (2003) p. 63, Zinsstag et al. (2005) .

7 Anderson (2004). The WHO defines ‘zoonosis’ as ‘diseases and infections which are naturally transmitted between man and animals’. World Health Organisation (1959) p. 6.

8 King (2004).

9 Jerolmack (2013).

10 Atlas and Maloy (2014), Zinsstag et al. (2015) .

11
*Infection Ecology & Epidemiology*—*The One Health Journal* (Co-Action Publishing, Sweden) was launched in 2011, while *One Health: The Official Journal of the OH Platform* (Elsevier) and *International Journal of One Health* (Veterinary World, Gujurat, India) were launched in 2015.

12 Kaplan and Scott (2011 ).

13 Nolan (2013).

14 These differences persist. For example, see Gates Foundation (2013), BBSRC (2014), Barrett and Boulay  (2015).

15 Craddock and Hinchliffe (2015), Cassidy (2016).

16 Degeling et al. (2015).

17 Benson (2011).

18 Nicholls (2011), Molloy (2011).

19 Cassidy (2012), Calain (2013), Wilkinson (2013), Lupton (2015).

20 Kahn et al. (2012) p.1.

21 Kahn et al. (2015 ).

22 Lee and Brumme (2012) pp. 1–8.

23 Leboeuf (2011), Wood et al. (2012), Chien (2013).

24 Gibbs (2014), One Health Sweden (2014).

25 Cassidy (2016).

26 For Schwabe’s experiences, see Chapter 10.1007/978-3-319-64337-3_5, p. 172. For other examples of veterinary-medical tensions, see Chapters 10.1007/978-3-319-64337-3_2 and 10.1007/978-3-319-64337-3_3. Also see Bresalier et al. (2015).

27 Schwabe (2004) pp. 194–5.

28 Schwabe (1964) p. vii.

29 The Bellagio Centre Residency Program is still active. See Rockefeller Foundation (2017).

30 Monath et al. (2010), Kaplan and Scott (2011) .

31 The bibliographic databases Web of Science and PubMed (which index the contents of medical, natural science and humanities research journals) were searched for the phrase ‘one medicine’ in journal publications from 1900 to the present. Seven citations were found using the term ‘OM’ prior to Schwabe (1984).

32 Schmidt (1962). On animals in American space medicine at this time, see Bimm (2013).

33 Ravdin (1965), Allam (1966), Lechner (1968), Parish and Schwartzmann (1971), Cass (1973).

34 Hendricks et al. (2009).

35 Editorial (1975) p. 535.

36 Kaplan and Scott (2011 ).

37 While Schwabe never held a position at the University of Pennsylvania, after his retirement he moved from California to nearby Haverford, Pennsylvania, suggesting personal or professional connections to the area. Schwabe (2003), Nolan (2013).

38 Editorial (1975), Editorial (2005), Obituary (2017).

39 Examples include: King et al. (2008), Monath et al. (2010).

40 Examples include: the construction of Charles Darwin as a scientific icon for contemporary evolutionary biology (Rees 2009), and the epidemiologist John Snow as a historical hero of public health (Vandenbroucke et al. 1991).

41 Bresalier et al. (2015).

42 See Cassidy (2016) p. 221.

43 Weisz and Olszynko-Gryn (2010).

44 Majok and Schwabe (1996), Gordon and Schwabe (2004).

45 Schwabe (1979). For a full bibliography, see Schwabe (2004).

46 See all papers in *Preventive Veterinary Medicine* 62 (2004).

47 Wildlife Conservation Society (2015, 2016) .

48 Wolfe et al. (1998), Karesh, et al. (2002), Wildlife Conservation Society (2016) .

49 AHEAD stands for Animal and Human Health for the Environment and Development. This global network is still active. For the earliest usage of OH, see Osofsky et al. (2003) p. 63.

50 For example: Cook et al. (2004), Karesh and Cook (2009) pp. 259–60.

51 Osofsky (2005) p. 83. For further details on ecosystem health, see for example Rapport et al. (1998).

52 See Chapter 10.1007/978-3-319-64337-3_5, p. 188.

53 For examples of the OWOH logo, see Cook et al. (2004).

54 Zinsstag et al. (2005 ).

55 Tanner (2005) pp. 403–404, World Health Organisation (2007) p. 2.

56 Zinsstag et al. (2005 ).

57 Rudolf was a member of the Geigy family, of the pharmaceutical and chemical company J.R. Geigy, inventors of dichlorodiphenyltrichloroethane (DDT), and now part of the contemporary Swiss pharmaceutical firm Novartis AG (Meier 2017).

58 See Chapter 10.1007/978-3-319-64337-3_5, also Worboys (1996).

59 Meier (2014, 2015).

60 The CSRS was administered by the Swiss Academy of Sciences until 2001, after which it was gradually handed over to Ivoirian scientists and national government.

61 For an overview of the group’s work, see Zinsstag (2017 ).

62 For example: Allen-Scott et al. (2015).

63 Zinsstag et al. (2012).

64 Mahr (2006), Enserink (2007).

65 Kahn and Davis (2008).

66 Kahn (2006).

67 Monath et al. (2010).

68 Kahn et al. (2007), Kaplan et al. (2009), Kahn et al. (2015).

69 King et al. (2008).

70 See, for example, the banner image used by Kahn et al. (2012).

71 King et al. (2008), Day (2010).

72 For further discussions of pandemic science and policy, and its tendency to orient towards publics in the developed world, see, for example, Caduff (2013).

73 Jerolmack (2013).

74 FAO, WHO, OIE et al. (2008).

75 FAO, OIE and WHO (2010).

76 Fujimura (1996, 1998).

77 Maye and Kirwan (2013), Bensaude Vincent (2014), Cassidy (2016).

78 Twine (2013), Hobson-West and Timmons (2016).

79 Gunelius (2010), Ecohealth Alliance (2017).

80 For example: Davies et al. (2016), Mwangi et al. (2016).

81 Cairns and Krzywoszynska (2016).

82 Source: Web of Science (Science, Social Science and Humanities Indices), excluding conference proceedings and incidental usage such as ‘one health authority’.

83 Galaz et al. (2015), Cassidy (2016). See also Friese and Nuyts (2017).

84 Manlove et al. (2016).

85 Bovine tuberculosis, caused by the bacterium M. bovis, is a zoonotic form of tuberculosis which can infect all mammal species, caused by the bacterium *M. bovis*. Brucellosis is a bacterial infection which causes abortion in cattle and fever and wasting in humans. Both infections are passed from animals to humans via contaminated meat and milk.

86 Davies (2012), Cassidy (2016).

87 Leptospirosis is a zoonotic bacterial infection carried primarily by rodents which can be fatal in severe cases. Rift Valley Fever and Hendra are both zoonotic viruses carried by livestock animals with a wildlife reservoir in bat populations.

88 Clarke and Fujimura (1992), Waddington (2006), Pemberton and Worboys (2007). See also Appendix: Annotated Bibliography of Animals in the History of Medicine.

89 Woods (2017).

90 See, for example, the banner logo of the OH Commission and that designed for the annual OH Day, initiated in 2016 (One Health Commission 2017) or that of the OH Platform and journal (One Health Platform 2017).

91 Osovsky et al. (2003).

92 See, for example, the logo of the EU Veterinary Week 2010 conference. EU Vet Week (2010).

93 For further analysis and commentary on the Blue Marble image, see Gurevitch (2014), Höhler (2015).

94 See the covers of Osofsky et al. (2003) and King et al. (2008).

95 One Health Global Network (2017).

96 For a fuller analysis of this kind of human-paw imagery, see Haraway (1999) pp. 134–9.

97 Examples include the first edition cover of David Quammen’s popular science book *Spillover* (2013), depicting a baboon baring its fangs; and the process of creation of a ‘scarier looking’ computer-generated imagery (CGI) bat for the 2011 film *Contagion* (Failes 2011).

98 Hernendez (2017).

99 Washer (2010), Cassidy (2012).

100 See, for example, Ducrotoy et al. (2015), Hinchliffe et al. (2016).

101 Berger (1980).

102 Fudge (2006).

103 Marks (2014), Okello et al. (2011), Cork et al. (2016).

104 Stephen and Karesh (2014), Galaz et al. (2015).

105 World Health Organisation (2015, 2016).
